# Novel Sulfamide-Containing Compounds as Selective Carbonic Anhydrase I Inhibitors

**DOI:** 10.3390/molecules22071049

**Published:** 2017-06-24

**Authors:** Emanuela Berrino, Silvia Bua, Mattia Mori, Maurizio Botta, Vallabhaneni S. Murthy, Vijayaparthasarathi Vijayakumar, Yasinalli Tamboli, Gianluca Bartolucci, Alessandro Mugelli, Elisabetta Cerbai, Claudiu T. Supuran, Fabrizio Carta

**Affiliations:** 1 NEUROFARBA Department, Sezione di Scienze Farmaceutiche e Nutraceutiche, Università degli Studi di Firenze, Via Ugo Schiff 6, 50019 Sesto Fiorentino (Florence), Italy; emanuela.berrino@unifi.it (E.B.); silvia.bua@unifi.it (S.B.); gianluca.bartolucci@unifi.it (G.B.); 2 Department of Biotechnology, Chemistry and Pharmacy, University of Siena, viale Aldo Moro 2, 53100 Siena, Italy; m.mattia79@gmail.com (M.M.); botta.maurizio@gmail.com (M.B.); 3 Sbarro Institute for Cancer Research and Molecular Medicine, Center for Biotechnology, College of Science 1.and Technology, Temple University, BioLife Science Building, Suite 333, 1900 N 12th Street, Philadelphia, PA 19122, USA; 4 Center for Organic and Medicinal Chemistry, School of Advanced Sciences, VIT University, Vellore, 632014 Tamil Nadu, India; murthychem@gmail.com (V.S.M.); kvpsvijayakumar@gmail.com (V.V.); 5 Department of Neurosciences, Psychology, Drug’s Research and Child’s Health (NEUROFARBA), University of Florence, Italy; Section of Pharmacology and Toxicology, viale Pieraccini 6, 50100 Firenze, Italy; alessandro.mugelli@unifi.it (A.M.); elisabetta.cerbai@unifi.it (E.C.)

**Keywords:** carbonic anhydrase inhibitors (CAIs), sulfamides, structure-activity-relationship (SAR)

## Abstract

The development of isoform selective inhibitors of the carbonic anhydrase (CA; EC 4.2.1.1) enzymes represents the key approach for the successful development of druggable small molecules. Herein we report a series of new benzenesulfamide derivatives (-NH-SO_2_NH_2_) bearing the 1-benzhydrylpiperazine tail and connected by means of a β-alanyl or nipecotyl spacer. All compounds **6a**–**l** were investigated in vitro for their ability to inhibit the physiological relevant human (h) CA isoforms such as I, II, IV and IX. Molecular modeling provided further structural support to enzyme inhibition data and structure-activity relationship. In conclusion the hCA I resulted the most inhibited isoform, whereas all the remaining ones showed different inhibition profiles.

## 1. Introduction

The carbonic anhydrases (CAs, EC 4.2.1.1) are ubiquitous enzymes belonging to the superfamily of metalloenzymes [1,2,3]. To date, fifteen isoforms of these enzymes have been reported in humans, and they all differ for kinetic properties, sub-cellular localization and tissue distribution [1,2]. They all catalyze a simple as well as critical reaction, namely the reversible conversion of carbon dioxide to bicarbonate and protons [1,2]. These small molecules (carbon dioxide, protons and bicarbonate) are also involved as natural substrates of many other enzymes of particular interest, such as sodium–bicarbonate co-transporters (NBCs), sodium–proton exchangers (NHEs) or chloride–bicarbonate exchanger (AEs) [4,5,6]. Thus, CA enzymes are deeply involved in several physiological pathways and any disruption of their activities may result in physiological dysfunctions [1,2,3]. Therefore the ability to modulate the CA’s enzymatic activities by means of the use of small molecules acting as inhibitors or activators may give access to the pharmacological treatment of human diseases [7,8,9]. 

**Figure 1:**
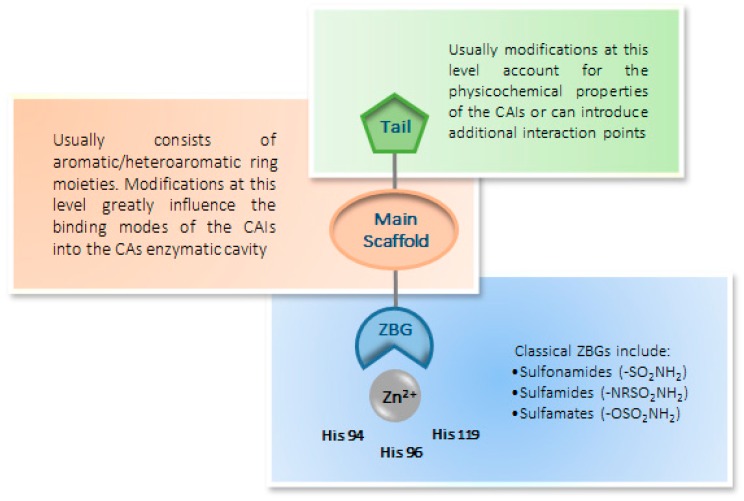
The ring and the tail approaches used for the specific inhibition of the carbonic anhydrases.

To date a large number of compounds have been explored and/or used as CA inhibitors (CAIs), and many of them exert their activity through different mechanisms [3,10,11,12,13]. Among them, the primary sulfonamide (R-SO_2_NH_2_)-containing compounds still represent the main class of CAIs explored, along with their bioisosteric analogs such as the sulfamides (-NH-SO_2_NH_2_) [14]. As reported from CA-ligand adduct X-ray crystallographic investigations the sulfamide moiety, when compared to the binding modes of the sulfonamides, ensures further interactions within the enzymatic cleft due to the presence of the additional nitrogen atom [3,14]. Despite the supplementary interaction points offered from the sulfamides within the CA, such a structural feature per se does not lead to selective isozyme binding [14]. Thus alternative design approaches have been developed with the aim to address the lack of selectively profiles associated to the CAIs of this type, and among others the tail approach is the most versatile (Figure 1) [14,15]. 

As schematically reported above, such an approach takes advantage from the ability of the tail moieties of the ligand to specifically interact with the amino acid residues present at the rim of the enzyme cavity, which is the most variable among the various enzyme isoforms [14,15]. 

Pursuing this strategy, we have synthesized a new series of sulfamides compounds **6a**–**l** bearing the 1-benzhydrylpiperazine tail and connected by means of a β-alanyl or nipecotyl spacer. All the obtained compounds then have been tested in vitro for their enzymatic activity on the dominant cytosolic physiological isoforms (hCA I, II), on the membrane-bound isoform hCA IV and on the transmembrane isoform hCA IX. 

## 2. Results and Discussion

### 2.1. Chemistry

**Scheme 1:**
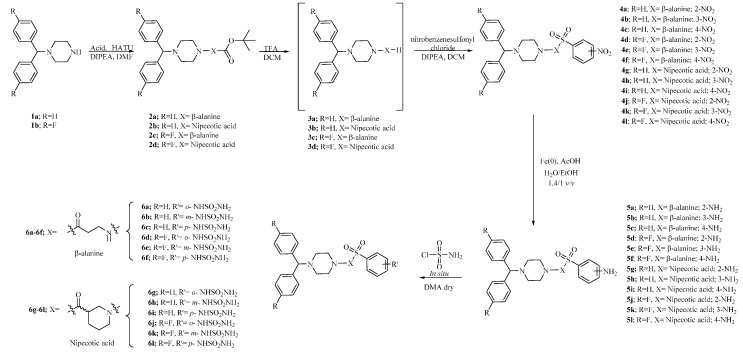
General synthetic scheme of compounds **6a**–**l**.

The aim of this study was to explore whether compounds bearing a sulfonamide bioisoster, such as the sulfamide (-NHSO_2_NH_2_) and installed into highly flexible alkyl-aryl scaffolds might show a significant enhancement of their selectivity profiles against the hCAs herein considered (I, II, IV and IX). In particular, we designed two series of compounds which differ in the spacer connecting to the 1-benzhydrylpiperazin tail with the sulfamide zinc binding group (Scheme 1). 

The intermediates **2a**–**d** were obtained by coupling of the benzhydryl piperazines **1a**,**b** with the appropriate acids [16,17], followed by treatment with TFA to afford the alkylamines **3a**–**d**. Then the free amines were coupled with commercially available nitrobenzenesulfonyl chlorides and the thus obtained nitro derivatives **4a**–**l** were reduced with Fe(0) in acidic media [18], to afford the amino derivatives **5a**–**l**. The desired compounds **6a**–**l** were obtained by treatment with freshly prepared sulfamoyl chloride [19]. All final compounds as well as their intermediates were characterized by means of ^1^H-, ^13^C-, ^19^F-NMR spectroscopy and HRMS, and were >95% pure as determined by HPLC.

### 2.2. Carbonic Anhydrase Inhibition

The final compounds **6a**–**l** were investigated for their ability to inhibit the main physiological relevant hCAs (I, II, IV and IX) by means of the stopped flow CO_2_ hydrase assay [20]. Inhibition data, compared to those of the standard sulfonamide inhibitor acetazolamide (AAZ), are reported in Table 1.

In general all compounds tested showed high-medium K_I_ inhibition values spanning between 45.8 and >10,000 nM. The following structure-activity-relationship (SAR) can be drawn:

(i) The hCA I was the most inhibited isoform among those considered in this study, with K_I_s in the range of 45.8–659.6 nM, and **6j** with a K_I_ value of 2750.9 nM (Table 1). SAR analyses showed that among the compounds having the β-alanyl spacer (**6a**–**c**), the inhibition potencies were strictly dependent from the sulfamide regioisomer considered. In particular, as reported in Table 1, the *meta*- and the *para*-sulfamide derivatives **6b** and **6c** resulted in three to four time greater potencies than the *ortho*-sulfamide **6a**. The introduction of a fluorine atom in **6a**–**c** to afford the corresponding derivatives **6d**–**f**, didn’t spoil the regioisomer-dependent inhibition trend. As reported in Table 1 the *meta*- and *para*-sulfamide fluoro containing derivatives **6e** and **6f** resulted 7 and 10 times more potent, respectively when compared to the *ortho*-regioisomer **6d** (K_I_s of 94.0, 63.2 and 659.6 nM respectively). The kinetic data relative to each regioisomer **6a**–**c**, when compared to its fluorinated derivative **6d**–**f**, showed that the introduction of the halogen was slightly detrimental for the inhibition activity (as for **6a** to **6d** and **6b** to **6e**). Conversely the *para* regioisomer **6f** showed a K_I_ value modestly reduced when compared to its non-halogenated counterpart (compound **6c**). As for the conformational restricted analogs **6g**–**l** a different regioisomer inhibition trend was reported (Table 1). Within both the non-halogenated (compounds **6g**–**i**) and halogenated (compounds **6j**–**l**) series, the placement of the sulfamide moiety in *ortho*- and *para*-position of the phenyl ring was clearly detrimental for the inhibition potency when compared to the corresponding *meta*-analogs. In analogy, the introduction of fluorine in **6g**–**i** to afford **6j**–**l**, further enhanced the inhibition K_I_ values of the *ortho*- and *para*-sulfamide derivatives (**6g** to **6j** and **6i** to **6l** respectively). On the contrary the fluorination of the *meta*-regioisomer **6h** to afford compound **6k** determined a 1.6 fold increase of the inhibition potency, thus making it as the most potent inhibitor of the hCA I among the series here considered.
(ii) As for the hCA II, all compounds herein considered showed medium-high K_I_ inhibition values and comprised between 89.8 and 6456.0 nM (Table 1). In general most of compounds bearing a conformational restricted spacer moiety, as in **6g**–**l**, showed higher inhibition K_I_ values when compared to their corresponding flexible analogs **6a**–**f** (Table 1). Conversely the compound **6g** resulted in a 5.3 fold potency increase when compared to its corresponding unrestricted analog **6a** (K_I_ 89.8 and 472.8 nM respectively). The introduction of the fluoro moiety within **6a**–**c** to afford the derivatives **6d**–**f**, determined an increase of the inhibition potencies of 2.5 fold for **6d** and **6e** and 1.07 fold for compound **6f** respectively. Conversely, among compounds **6g**–**l** the fluorination resulted detrimental for the inhibition activity against the hCA II. A 72.5 fold increase of the K_I_ value was obtained for compound **6j** when compared to its non-halogenated counterpart. In analogy the fluorination slightly spoiled the inhibition potency also for **6l**, which was 1.7 fold less potent than **6i**. Conversely a slight potency improvement (1.2-fold) was observed for the *meta*-fluorinated derivative **6k** when compared to its analog **6h**. The K_I_ inhibition values among the non-fluorinated compounds **6a**–**c** and **6g**–**i** resulted particularly affected from the sulfamide ZGB regioisomers. As reported in Table 1 the potency ranking for compounds **6a**–**c** was *meta* > *para* > *ortho*, whereas the constrained analogs **6g**–**i** showed the opposite trend. As for the fluorinated derivatives **6d**–**f** and **6j**–**l**, the *meta*-substituted compounds **6e** and **6k** were still the most potent, however a switch between the *para* and *ortho* substituted derivatives was observed.
(iii) In analogy to the hCA I isoform, the inhibition data on compounds **6a**–**l** on the hCA IV, revealed a potency decrease for the conformational restricted series **6g**–**l** when compared with their flexible analogs **6a**–**f**, with the only exception represented by the *meta*-sulfamide substituted compound **6h** (Table 1). Among the conformationally unrestricted series **6a**–**f**, the introduction of the fluorine moiety on compound **6a** and **6b**, to afford **6d** and **6e**, determined a 1.3- and 5.6- fold increase, respectively, of the inhibition potency. On the other hand the same substitution within **6c** to afford **6f** resulted detrimental (1.6 fold) for their kinetic potency.
  Among the conformationally restricted series **6g**–**l** the introduction of the fluoro moiety resulted detrimental for the inhibition potency of the *ortho* and *para* derivatives (compounds **6j** and **6l** respectively). The meta-sulfamide substituted derivative **6k** resulted slightly more potent when compared to its corresponding non-fluorinated counterpart **6h** (1.2-fold).
(iv) The tumor associated isoform hCA IX was poorly inhibited by the compounds herein reported with K_I_s spanning between 2682.4 and 216.7 nM, whereas compound **6a** and its conformationally restricted derivative **6g** were ineffective (K_I_ > 10,000 nM). Interestingly the fluorination resulted in a clear enhancement of the inhibition activities. Noteworthy when the fluorine moiety was introduced within compounds **6a** and **6g**, to afford **6d** and **6j** respectively, the inhibition activity was restored (K_I_s of 735.1 and 1233.3 nM respectively). SAR evaluation within the **6a**–**f** series showed that the *meta*-sulfamide substituted derivatives **6b** and **6e** were more potent when compared to their corresponding regioisomers. The same kinetic trend was also observed within the fluoro-substituted constrained derivatives **6j**–**l**, and not for their non-fluorinated counterparts **6g**–**i** (Table 1). Interestingly the *meta* derivatives **6e** and **6k** were the most potent inhibitors against the hCA IX among the series here considered (K_I_ 216.7 and 296.5 nM respectively).

### 2.3. Molecular Modeling

To decipher the possible binding mode of hCA I inhibitors studied herein, and to provide a structural support to the SAR above discussed, molecular modeling studies were conducted. Thanks to the availability of the crystallographic structure of hCA I isoform, molecular docking simulations were performed on a representative subset of sulfamides **6a**–**l**. In particular, **6c**, **6e** and **6f** bearing the β-alanine spacer were selected to monitor the influence of the sulfamide regioisomer on the binding mode, as well as the possible role of fluorine atoms. Compound **6k** was selected as it showed the strongest inhibition value for hCA I among the test set, and bears a conformationally restrained linker. It is worth mentioning that both enantiomers of **6k** were modeled and provided comparable poses; however, only the *R*-enantiomer is discussed due to its higher agreement with SAR. Based on prior structural data, [21,22,23,24] a covalent docking approach was carried out with the GOLD program (version 5.2.2) [25,26] to link directly the terminal nitrogen atom of the sulfamide group to the catalytic Zn(II) ion in molecular docking simulations. 

**Figure 2:**
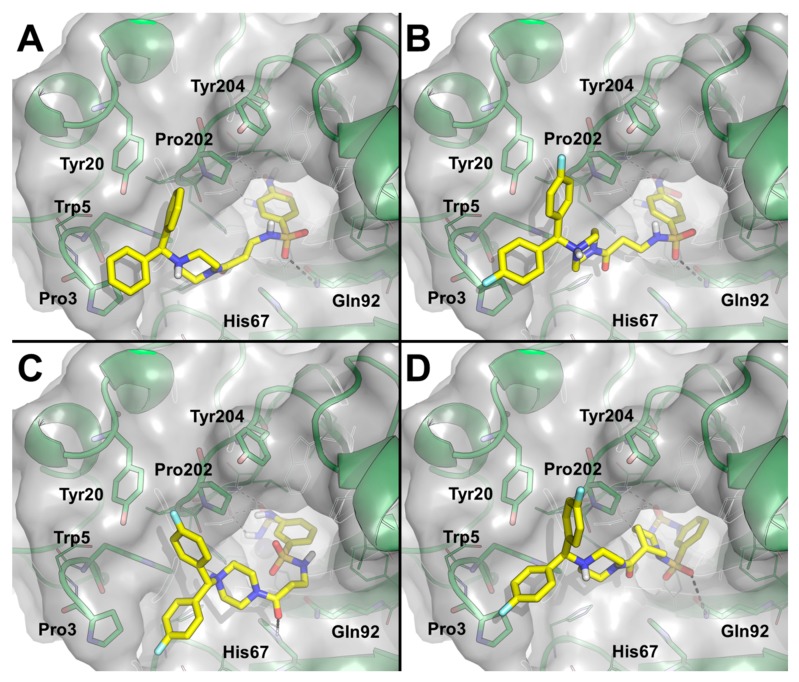
Predicted binding mode of compounds **6c** (**A**), **6f** (**B**), **6e** (**C**) and **6k** (**D**). Small molecules are showed as yellow sticks, non-polar hydrogen atoms are omitted. The crystallographic structure of hCA I (PDB ID: 4WR7) is shown as green cartoon and grey transparent surface. Residues within 5 Å from the ligands are showed as green lines, residues contacted by the inhibitors and described in the text are showed as sticks and labeled. The catalytic Zn(II) ion is shown as grey sphere. Polar interactions between inhibitors and hCA I are highlighted by dashed lines.

Consistent with the design strategy used in this work, the sulfamide core of **6c**, **6e**, **6f** and **6k** was well inserted inside the narrow hCA I catalytic cleft (Figure 2). Besides the constrained binding to the Zn(II) ion, the sulfamide moiety established the canonical H-bond interactions with Thr199 (residues numbering in agreement with the crystallographic structure). The phenyl ring occupies a position that is highly comparable with available structural data, [23] whereas the sulfamide group interacted with the side chain of Gln92 in **6c**, **6f** and **6k** (Figure 2A,B,D, respectively). This interaction did noit occur with **6e** because of the different orientation imposed by the *meta*-sulfamide moiety coupled with the β-alanyl linker, even if the high affinity for the enzyme was guaranteed in **6e** by the peculiar H-bond interaction between the linker’s carbonyl group and the His67 residue (Figure 2C). Whether *meta*- and *para*-sulfamides are allowed to fit the catalytic site, the *ortho*-substitution resulted sterically not allowed by molecular docking, and in agreement with experimental evidences and SAR (Table 1).

As expected from the rational design, the linker projects the tail portion of these hCA I inhibitors towards the rim of the catalytic site. This is a solvent-exposed region endowed with a high variability among CAs, and in hCA I is composed by a number of hydrophobic and aromatic residues such as Pro3, Trp5, Tyr20 (Figure 2) that are involved in binding to the inhibitors. The hydrophobic cleft that accommodates the aromatic tail of **6c**, **6e**, **6f** and **6k** is further complemented by residues Pro202 and Tyr204 (Figure 2). Overall, the tail of inhibitors **6c** and **6f** is docked in a highly superimposable manner (Figure 2A,B), thus suggesting that the introduction of fluorine does not impact on the binding to hCA I at a structural level. The weaker affinity of **6c** than **6f** could be explained by the higher hydrophobicity of the phenyl rings in **6c** compared to the fluorine derivatives in **6f**, particularly when occupying a solvent-exposed cleft. 

In the case of **6e**, the different sulfamide regioisomer determines a slightly different positioning of the tail moiety, which is more included in the catalytic tunnel than **6c** and **6f** (Figure 2C). Accordingly, fluorine atoms occupy an unfavorable region less exposed to the solvent, as also supported by the slightly higher efficacy of the non-fluorinated analogue **6b** (Table 1). Moreover, the protonated NH group of the piperazine moiety of **6e** points towards the surface of the protein, whereas in all other compounds it is exposed to the solvent, that further explains the relatively lower affinity of **6e**. 

Finally, the conformationally restrained **6k** shares a similar binding conformation with **6c** and **6f** (Figure 2D) even if they bear a different sulfamide regioisomer. The combination between *meta*-sulfamide zinc-binding group and a restrained linker provides the stronger inhibition of hCA I. Also in this compound, the solvent exposed fluorine atoms provide a noticeable gain of hCA I inhibition with respect to the unsubstituted phenyl ring in the analogue **6h** (Table 1). 

## 3. Experimental Protocols

### 3.1. Chemistry

Anhydrous solvents and all reagents were purchased from Sigma-Aldrich (Milan, Italy), Alfa Aesar (Milan, Italy) and TCI (Milan, Italy). All reactions involving air- or moisture-sensitive compounds were performed under a nitrogen atmosphere using dried glassware and syringes techniques to transfer solutions. Nuclear magnetic resonance spectra (^1^H-NMR: 400 MHz; ^13^C-NMR: 100 MHz; ^19^F-NMR: 376 MHz) were recorded in DMSO-*d*_6_ using an Avance III 400 MHz spectrometer (Bruker, Milan, Italy). Chemical shifts are reported in parts per million (ppm) and the coupling constants (*J*) are expressed in Hertz (Hz). Splitting patterns are designated as follows: s, singlet; d, doublet; t, triplet; q, quadruplet; m, multiplet; brs, broad singlet; dd, double of doublets. The assignment of exchangeable protons (OH and NH) was confirmed by the addition of D_2_O. Analytical thin-layer chromatography (TLC) was carried out on silica gel F-254 plates (Merck, Milan, Italy). Melting points (m.p.) were carried out in open capillary tubes and are uncorrected. The solvents used in MS measures were acetone, acetonitrile (Chromasolv grade), purchased from Sigma–Aldrich and mQ water 18 MX, obtained from Millipore’s Simplicity system (Milan, Italy). The mass spectra were obtained using a 1200 L triple quadrupole system (Varian, Palo Alto, CA, USA) equipped by Electrospray Source (ESI) operating in both positive and negative ions. Stock solutions of analytes were prepared in acetone at 1.0 mg mL^−1^ and stored at 4 ^°^C. Working solutions of each analyte were freshly prepared by diluting stock solutions in a mixture of mQ H_2_O/ACN 1:1 (*v*/*v*) up to a concentration of 1.0 μg mL^−1^. The mass spectra of each analyte were acquired by introducing, via syringe pump at 10 μL min^−1^, of the its working solution. Raw-data were collected and processed by Varian Workstation Vers. 6.8 software.

#### 3.1.1. General Procedure for the Synthesis of Compounds **2a**–**d**

Compounds **1a**,**b** (1.0 eq) and the appropriate *N*-Boc-protected carboxylic acid (1.1 eq) in DMF (10.0 mL) were treated with DIPEA (2.0 eq), and HATU (1.5 eq) at r.t. for 30 min. When the reaction was complete (TLC monitoring), it was quenched with slush and extracted with ethyl acetate (3 × 15 mL). The combined organic layers were washed with H_2_O (3 × 15 mL), dried over Na_2_SO_4_, filtered-off and concentrated under reduced pressure to afford the title compounds **2a**–**d** [17] as white solids.

*tert-Butyl (3-(4-benzhydrylpiperazin-1-yl)-3-oxopropyl)carbamate* (**2a**). Using **1a** and *N*-Boc-β-alanine as starting materials and the general procedure described above compound **2a** was obtained in 95% yield. ^1^H-NMR: δ 1.12 (9H, s, 3 × CH_3_), 2.26 (4H, m, 2 × piperazine-CH_2_), 2.47 (2H, t, *J* = 7.2, COCH_2_), 3.08 (2H, m, CH_2_NH), 3.38 (4H, m, 2 × piperazine-C*H*_2_), 4.30 (1H, s, CH), 7.17 (2H, appt, *J* = 7.4, Ar-*H*), 7.28 (4H, appt, *J* = 7.4, Ar-*H*), 7.41 (4H, d, *J* = 7.4, Ar-*H*), 7.6 (1H, brs, NH).

*tert-Butyl 3-(4-benzhydrylpiperazine-1-carbonyl)piperidine-1-carboxylate* (**2b**). Using **1a** and *N*-Boc-nipecotic acid as starting materials compound **2b** was obtained in 80% yield. ^1^H-NMR: δ 1.13 (9H, s, 3 × CH_3_), 1.30 (1H, m, piperidine-CH), 1.50 (1H, m, piperidine-CH), 1.73 (2H, m, piperidine-CH_2_), 2.30 (4H, m, 2 × piperazine-CH_2_), 2.75 (3H, m, piperidine-CH_2_, COCH), 3.49 (4H, m, 2 × piperazine-CH_2_), 3.71 (2H, m, piperidine-CH_2_), 4.30 (1H, s, CH) 7.18 (2H, appt, *J* = 7.2, Ar-*H*), 7.29 (4H, appt, *J* = 7.2, Ar-*H*), 7.42 (4H, d, *J* = 7.2, Ar-*H*).

*tert-Butyl (3-(4-(bis(4-fluorophenyl)methyl)piperazin-1-yl)-3-oxopropyl)carbamate* (**2c**). Using **1b** and *N*-Boc-β-alanine as starting materials compound **2c** was obtained in 99% yield. ^1^H-NMR: δ 1.12 (9H, s, 3 × CH_3_), 2.26(4H, m, 2 × piperazine-CH_2_), 2.47 (2H, t, *J* = 7.2, COC*H*_2_), 3.08 (2H, m, CH_2_NH), 3.38 (4H, m, 2 × piperazine-CH_2_), 4.30 (1H, s, CH), 7.12 (4H, m, Ar-*H*), 7.42 (4H, m, Ar-*H*), 7.6 (1H, brs, NH).

*tert-Butyl 3-(4-(bis(4-fluorophenyl)methyl)piperazine-1-carbonyl)piperidine-1-carboxylate* (**2d**). Using **1b** and *N*-Boc-nipecotic acid are as starting materials compound **2d** was obtained in 95% yield. ^1^H-NMR: δ 1.13 (9H, s, 3 × CH_3_), 1.30 (1H, m, piperidine-CH), 1.50 (1H, m, piperidine-CH), 1.73 (2H, m, piperidine-CH_2_), 2.30 (4H, m, 2 × piperazine-CH_2_), 2.75 (3H, m, piperidine-CH_2_, COCH), 3.49 (4H, m, 2 × piperazine-CH_2_), 3.71 (2H, m, piperidine-CH_2_), 4.30 (1H, s, CH), 7.12 (4H, m, Ar-*H*), 7.42 (4H, m, Ar-*H*).

#### 3.1.2. General Procedure for the Synthesis of Compounds **4a**–**l**

A stirred solution of compounds **2a**–**d** (1.0 eq) in DCM (10.0 mL) was treated with TFA (3.0 eq) and stirred at r.t. for 2h. The reaction mixture was concentrated to dry and co-distilled twice with DCM to afford the corresponding alkyl amines **3a**–**d** as TFA salts (not isolated), which were readily dissolved in DCM (10.0 mL) and treated with DIPEA (5.0 eq) and the appropriate sulfonyl chloride (1.2 eq). The reaction solutions were stirred at r.t. for 1 h, then concentrated to dry and the residue obtained was purified by silica gel column chromatography using ethyl acetate in *n*-hexane (20–40% *v*/*v*) as eluents to afford the titled compounds **4a**–**l** as white solids.

*N-(3-(4-Benzhydrylpiperazin-1-yl)-3-oxopropyl)-2-nitrobenzenesulfonamide* (**4a**). Using **3a** and 2-nitro- benzenesulfonyl chloride as starting materials compound **4a** was obtained in 29% yield according to the general procedure described above; TLC: *R*_f_ = 0.39 (ethyl acetate/*n*-hexane 70% *v*/*v*); ^1^H-NMR: δ 2.22 (4H, m, 2 × piperazine-CH_2_), 2.45 (2H, t, *J* = 7.2, COCH_2_), 3.08 (2H, m, CH_2_NH), 3.38 (4H, m, 2 × piperazine-CH_2_), 4.28 (1H, s, CH), 7.17 (2H, appt, *J* = 7.4, Ar-*H*), 7.28 (4H, appt, *J* = 7.4, Ar-*H*), 7.41 (4H, d, *J* = 7.4, Ar-*H*), 7.91 (5H, m, overlapping signals; exchangeable with D_2_O, SO_2_NHCH_2_, 4 × Ar-*H*); ^13^C-NMR: δ 32.4, 41.0, 44.7, 51.1, 74.6, 124.4, 126.9, 127.5, 128.5, 129.5, 132.5, 132.7, 134.0, 142.4, 147.7, 168.2; MS (ESI) *m*/*z* = 508.9 [M + 1]^+^.

*N-(3-(4-Benzhydrylpiperazin-1-yl)-3-oxopropyl)-3-nitrobenzenesulfonamide* (**4b**). Using **3a** and 3-nitro-benzenesulfonyl chloride as starting materials compound **4b** was obtained in 56% yield; TLC: *R*_f_ = 0.28 (ethyl acetate/*n*-hexane 50% *v*/*v*); ^1^H-NMR: δ 2.23 (4H, m, 2 × piperazine-CH_2_), 2.42 (2H, t, *J* = 6.8, COC*H*_2_), 2.98 (2H, t, *J* = 6.8, C*H*_2_NH), 3.37 (4H, m, 2 × piperazine-C*H*_2_), 4.27 (1H, s, C*H*), 7.18 (2H, appt, *J* = 7.4, Ar-*H*), 7.28 (4H, appt, *J* = 7.4, Ar-*H*), 7.40 (4H, d, *J* = 7.4, Ar-*H*), 7.88 (1H, t, *J* = 7.6, Ar-*H*), 7.96 (1H, s, exchangeable with D_2_O, CH_2_N*H*SO_2_), 8.19 (1H, d, *J* = 7.6, Ar-*H*), 8.46 (1H, d, *J* = 7.6, Ar-*H*), 8.51 (1H, s, Ar-*H*); ^13^C-NMR: δ 32.4, 38.8, 44.7, 51.0, 74.7, 121.3, 126.8, 126.9, 127.5, 128.5, 131.2, 132.5, 142.1, 142.4, 147.9, 168.7; MS (ESI) *m*/*z* = 508.9 [M + 1]^+^.

*N-(3-(4-Benzhydrylpiperazin-1-yl)-3-oxopropyl)-4-nitrobenzenesulfonamide* (**4c**). Using **3a** and 4-nitro-benzenesulfonyl chloride as starting materials compound **4c** was obtained in 54% yield; TLC: *R*_f_ = 0.72 (ethyl acetate/*n*-hexane 70% *v*/*v*); ^1^H-NMR: δ 2.27 (4H, m, 2 × piperazine-CH_2_), 2.50 (2H, t, *J* = 6.8, COCH_2_), 3.05 (2H, t, *J* = 6.8, CH_2_NH), 3.37 (4H, m, 2 × piperazine-CH_2_), 4.32 (1H, s, CH), 7.24 (2H, appt, *J* = 7.4, Ar-*H*), 7.34 (4H, appt, *J* = 7.4, Ar-*H*), 7.46 (4H, d, *J* = 7.4, Ar-*H*), 8.03 (1H, s, exchangeable with D_2_O, CH_2_NHSO_2_), 8.08 (2H, d, *J* = 8.8, Ar-*H*), 8.46 (2H, d, *J* = 8.8, Ar-*H*); ^13^C-NMR: δ 32.4, 38.9, 44.6, 51.1, 74.7, 124.5, 126.9, 127.5, 128.0, 128.5, 142.4, 146.0, 149.4, 168.0; MS (ESI) *m*/*z* = 508.9 [M + 1]^+^.

*N-(3-(4-(bis(4-Fluorophenyl)methyl)piperazin-1-yl)-3-oxopropyl)-2-nitrobenzenesulfonamide* (**4d**). Using **3c** and 2-nitrobenzenesulfonyl chloride as starting materials compound **4d** was obtained in 53% yield; TLC: *R*_f_ = 0.30 (MeOH/DCM 10% *v*/*v*); ^1^H-NMR: δ 2.20 (4H, m, 2 × piperazine-CH_2_), 2.47 (2H, m, COCH_2_), 3.08 (2H, t, *J* = 6.8, CH_2_NH), 3.38 (4H, m, 2 × piperazine-CH_2_), 4.37 (1H, s, CH), 7.12 (4H, m, Ar-*H*), 7.42 (4H, m, Ar-*H*), 7.85–7.98 (5H, m, exchangeable with D_2_O, SO_2_N*H*CH_2_, 4 × Ar-*H*); ^13^C-NMR: δ 32.4, 39.1, 44.6, 50.8, 72.5, 116.2 (d, ^2^*J*_C–F_ 21), 124.5, 129.3, 130.3 (d, ^3^*J*_C–F_ 8), 132.7, 134.0, 138.3, 138.5, 147.7, 162.1 (d, ^1^*J*_C–F_ 242), 168.2; δ_F_ (376 MHz, DMSO-*d*_6_) −115.6 (2F, s); MS (ESI) *m*/*z* = 543.43 [M − 1]^+^.

*N-(3-(4-(bis(4-Fluorophenyl)methyl)piperazin-1-yl)-3-oxopropyl)-3-nitrobenzenesulfonamide* (**4e**). Using **3c** and 3-nitrobenzenesulfonyl chloride as starting materials compound **4e** was obtained in 66%; TLC: *R*_f_ = 0.25 (MeOH/DCM 5% *v*/*v*); ^1^H-NMR: δ 2.19 (4H, m, 2 × piperazine-CH_2_), 2.43 (2H, m, COCH_2_), 2.98 (2H, t, *J* = 6.8, CH_2_NH), 3.34 (4H, m, 2 × piperazine-C*H*_2_), 4.36 (1H, s, CH), 7.11 (4H, t, *J* = 9.0, Ar-*H*), 7.42 (4H, mAr-*H*), 7.88 (1H, t, *J* = 7.6, Ar-*H*), 7.96 (1H, s, exchangeable with D_2_O, CH_2_N*H*SO_2_), 8.19 (1H, d, *J* = 7.6, Ar-*H*), 8.46 (1H, d, *J* = 7.6, Ar-*H*), 8.51 (1H, s, Ar-*H*); ^13^C-NMR: δ 32.4, 38.8, 44.7, 51.4, 72.6, 116.0 (d, ^2^*J*_C–F_ 21), 121.4, 127.0, 130.3 (d, ^3^*J*_C–F_ 8), 132.6, 138.2, 138.4, 142.1, 147.9, 162.1 (d, ^1^*J*_C–F_ 242), 168.1; δ_F_ (376 MHz, DMSO-*d*_6_) −115.6 (2F, s); MS (ESI) *m*/*z* = 545.11 [M + 1]^+^.

*N-(3-(4-(bis(4-Fluorophenyl)methyl)piperazin-1-yl)-3-oxopropyl)-4-nitrobenzenesulfonamide* (**4f**). Using **3c** and 4-nitrobenzenesulfonyl chloride as starting materials compound **4f** was obtained in 48% yield; TLC: *R*_f_ = 0.48 (MeOH/DCM 5% *v*/*v*); ^1^H-NMR: δ 2.19 (4H, m, 2 × piperazine-CH_2_), 2.43 (2H, t, *J* = 6.8, COCH_2_), 2.98 (2H, q, *J* = 6.8, CH_2_NH), 3.36 (4H, m, 2 × piperazine-C*H*_2_), 4.36 (1H, s, CH), 7.12 (4H, t, *J* = 8.8, Ar-*H*), 7.41 (4H, mAr-*H*), 7.97 (1H, t, *J* = 5.6, exchangeable with D_2_O, CH_2_NHSO_2_), 8.02 (2H, d, *J* = 9.2, Ar-*H*), 8.40 (2H, d, *J* = 9.2, Ar-*H*); ^13^C-NMR: δ 32.6, 38.8, 44.6, 51.4, 72.6, 115.9 (d, ^2^*J*_C–F_ 21), 124.6, 128.1, 130.3 (d, ^3^*J*_C–F_ 8), 138.6, 146.0, 149.5, 162.0 (d, ^1^*J*_C–F_ 242), 168.1; ^19^F-NMR: δ −115.6 (2F, s); MS (ESI) *m*/*z* = 545.09 [M + 1]^+^.

*(4-Benzhydrylpiperazin-1-yl)(1-((2-nitrophenyl)sulfonyl)piperidin-3-yl)methanone* (**4g**). Using **3b** and 2-nitrobenzenesulfonyl chloride as starting materials compound **4g** was obtained in 31% yield; TLC: *R*_f_ = 0.40 (ethyl acetate/*n*-hexane 60% *v*/*v*); ^1^H-NMR: δ 1.30 (1H, m, piperidine-CH), 1.50 (1H, m, piperidine-CH), 1.73 (2H, m, piperidine-CH_2_), 2.30 (4H, m, 2 × piperazine-CH_2_), 2.75 (3H, m, piperidine-CH_2_, COCH), 3.49 (4H, m, 2 × piperazine-CH_2_), 3.71 (2H, m, piperidine-CH_2_), 4.30 (1H, s, CH) 7.18 (2H, appt, *J* = 7.2, Ar-*H*), 7.29 (4H, appt, *J* = 7.2, Ar-*H*), 7.42 (4H, d, *J* = 7.2, Ar-*H*) 7.90 (2H, m, Ar-*H*), 8.02 (2H, m, Ar-*H*); ^13^C-NMR: δ 23.9, 26.6, 41.0, 44.7, 45.8, 47.9, 51.9, 74.6, 124.1, 126.9, 127.5, 128.5, 129.6, 130.1, 132.2, 134.6, 142.4, 147.7, 170.2 ; MS (ESI) *m*/*z* = 548.9 [M + 1]^+^.

*(4-Benzhydrylpiperazin-1-yl)(1-((3-nitrophenyl)sulfonyl)piperidin-3-yl)methanone* (**4h**). Using **3b** and 3-nitrobenzenesulfonyl chloride as starting materials compound **4h** was obtained in 47% yield; TLC: *R*_f_ = 0.47 (ethyl acetate/*n*-hexane 60% *v*/*v*); ^1^H-NMR: δ 1.29 (1H, m, piperidine-CH), 1.52 (1H, m, piperidine-CH), 1.73 (2H, m, piperidine-CH_2_), 2.30 (4H, m, 2 × piperazine-CH_2_), 2.71 (1H, m, COCH), 2.86 (2H, m, piperidine-CH_2_), 3.49 (4H, m, 2 × piperazine-CH_2_), 3.71 (2H, m, piperidine-C*H*_2_), 4.31 (1H, s, CH), 7.18 (2H, appt, *J* = 7.4, Ar-*H*), 7.30 (4H, appt, *J* = 7.4, Ar-*H*), 7.43 (4H, d, *J* = 7.4, Ar-*H*), 7.96 (1H, t, *J* = 8.8, Ar-*H*), 8.19 (1H, d, *J* = 8.8, Ar-*H*), 8.39 (1H, s, Ar-*H*); 8.55 (1H, d, *J* = 8.8, Ar-*H*); ^13^C-NMR: δ 23.6, 26.6, 41.0, 44.9, 45.9, 48.0, 51.9, 74.7, 121.8, 126.9, 127.5, 127.6, 127.7, 128.6, 131.6, 133.3, 142.5, 148.1, 170.3; MS (ESI) *m*/*z* = 548.9 [M + 1]^+^.

*(4-Benzhydrylpiperazin-1-yl)(1-((4-nitrophenyl)sulfonyl)piperidin-3-yl)methanone* (**4i**). Using **3b** and 4-nitrobenzenesulfonyl chloride compound **4i** was obtained in 45% yield; TLC: *R*_f_ = 0.33 (ethyl acetate/*n*-hexane 40% *v*/*v*); ^1^H-NMR: δ 1.22 (1H, m, piperidine-CH), 1.53 (1H, m, piperidine-CH), 1.69 (2H, m, piperidine-CH_2_), 2.32 (6H, m, 2 × piperazine-CH_2_, piperidine-CH_2_), 2.80 (1H, m, COCH), 3.49 (4H, m, 2 × piperazine-CH_2_), 3.54 (2H, m, piperidine-CH_2_), 4.30 (1H, s, CH), 7.18 (2H, appt, *J* = 7.4, Ar-*H*), 7.30 (4H, appt, *J* = 7.4, Ar-*H*), 7.43 (4H, d, *J* = 7.4, Ar-*H*), 7.99 (2H, d, *J* = 9.0), 8.43 (2H, d, *J* = 9.0); ^13^C-NMR: δ 23.6, 26.6, 41.0, 44.8, 45.9, 48.1, 51.9, 74.6, 124.7, 126.9, 127.5, 128.5, 128.8, 141.3, 142.4, 149.9, 170.3; MS (ESI) *m*/*z* = 548.9 [M + 1]^+^.

*(4-(bis(4-Fluorophenyl)methyl)piperazin-1-yl)(1-((2-nitrophenyl)sulfonyl)piperidin-3-yl)methanone* (**4j**). Using **3d** and 2-nitrobenzenesulfonyl chloride as starting materials compound **4j** was obtained in 57% yield; TLC: *R*_f_ = 0.52 (ethyl acetate/*n*-hexane 70% *v*/*v*); ^1^H-NMR: δ 1.25 (1H, m, piperidine-CH), 1.50 (1H, m, piperidine-CH), 1.74 (2H, m, piperidine-CH_2_), 2.26 (4H, m, 2 × piperazine-CH_2_), 2.63 (1H, m, COCH), 2.75 (2H, m, piperidine-CH_2_), 3.46 (4H, m, 2 × piperazine-CH_2_), 3.64 (2H, m, piperidine-CH_2_), 4.41 (1H, s, CH), 7.12 (4H, t, *J* = 8.4, Ar-*H*), 7.43 (4H, m, Ar-*H*), 7.87 (4H, m, Ar-*H*); ^13^C-NMR: δ 23.9, 26.6, 41.0, 44.7, 45.7, 47.8, 51.7, 72.5, 115.2, 116.2 (d, ^2^*J*_C–F_ 21), 129.3, 129.4, 129.9, 131.1 (d, ^3^*J*_C–F_ 9), 134.6, 138.3, 147.7, 162.1 (d, ^1^*J*_C–F_ 242), 170.2; ^19^F-NMR: δ −115.6 (2F, s); MS (ESI) *m*/*z* = 585.24 [M + 1]^+^.

*(4-(bis(4-Fluorophenyl)methyl)piperazin-1-yl)(1-((3-nitrophenyl)sulfonyl)piperidin-3-yl)methanone* (**4k**). Using **3d** and 3-nitrobenzenesulfonyl chloride as starting materials compound **4k** was obtained in 68% yield; TLC: *R*_f_ = 0.53 (ethyl acetate/*n*-hexane 70% *v*/*v*); ^1^H-NMR: δ 1.17 (1H, m, piperidine-CH), 1.55 (1H, m, piperidine-CH), 1.69 (2H, m, piperidine-CH_2_), 2.26 (6H, m, 2 × piperazine-CH_2_, piperidine-CH_2_), 2.80 (1H, m, COCH), 3.63(6H, m, 2 × piperazine-CH_2_, piperidine-CH_2_), 4.41 (1H, s, C*H*), 7.12 (4H, t, *J* = 8.4, Ar-*H*), 7.43 (4H, m, Ar-*H*), 7.96 (1H, t, *J* = 8.8, Ar-*H*), 8.16 (1H, d, *J* = 8.8, Ar-*H*), 8.35 (1H, m, Ar-*H*); 8.55 (1H, d, *J* = 8.8, Ar-*H*); ^13^C-NMR: δ 23.6, 26.6, 41.1, 44., 45.9, 48.0, 51.8, 72.6, 115.3 (d, ^2^*J*_C–F_ 21), 121.8, 129.3, 129.4, 129.4, 130.5 (d, ^3^*J*_C–F_ 8, 133.3), 137.5, 138.4, 148.1, 161 (d, ^1^*J*_C–F_ 242), 170.3; ^19^F-NMR: δ −115.6 (2F, s) MS (ESI) *m*/*z* = 585.14 [M + 1]^+^.

*(4-(bis(4-Fluorophenyl)methyl)piperazin-1-yl)(1-((4-nitrophenyl)sulfonyl)piperidin-3-yl)methanone* (**4l**). Using **3d** and 4-nitrobenzenesulfonyl chloride as starting materials compound **4l** was obtained in 57% yield; TLC: *R*_f_ = 0.84 (ethyl acetate/*n*-hexane 70% *v*/*v*); ^1^H-NMR: δ 1.15 (1H, m, piperidine-CH), 1.55 (1H, m, piperidine-CH), 1.69 (2H, m, piperidine-CH_2_), 2.26 (6H, m, 2 × piperazine-CH_2_, piperidine-CH_2_), 2.81 (1H, m, COCH), 3.56 (6H, m, 2 × piperazine-CH_2_, piperidine-CH_2_), 4.39 (1H, s, CH), 7.12 (4H, t, *J* = 8.4, Ar-*H*), 7.43 (4H, m, Ar-*H*), 7.98 (2H, d *J* = 9.2, Ar-*H*) 8.42 (2H, d *J* = 9.2, Ar-*H*); ^13^C-NMR: δ 23.6, 26.6, 41.0, 44.7, 45.8, 48.0, 51.7, 72.5, 115.3 (d, ^2^*J*_C–F_ 21), 124 .6, 128.9, 129.3 (d, ^3^*J*_C–F_ 9), 138.2, 141.4, 149.9, 162.0 (d, ^1^*J*_C–F_ 24), 170.2; MS (ESI) *m*/*z* = 585.15 [M + 1]^+^.

#### 3.1.3. General Procedure for the Synthesis of Amino Benzensulfonamides **5a**–**l**

The appropriate nitrobenzenesulfonamides **4a**–**l** (1.0 eq) in a solution of H_2_O (0.4 mL) and EtOH (0.3 mL) was treated with glacial AcOH (0.05 mL) and Fe (0) (12.0 eq). The reaction mixture was stirred at 75 °C for 1 h (TLC monitoring), then cooled to r.t. and diluted with EtOAc (10.0 mL). The mixture was filtered through Celite 521^®^, washed with a saturated NaHCO_3_ aqueous solution (3 × 15 mL), brine (3 × 10 mL) and dried over Na_2_SO_4_. The organic solvent was evaporated *in vacuo* to give an oil residue, which was triturated from Et_2_O, to afford the titled compounds **5a**–**l** [18] as white solids.

*2-Amino-N-(3-(4-benzhydrylpiperazin-1-yl)-3-oxopropyl)benzenesulfonamide* (**5a**). Compound **5a** was obtained in 80% yield; m.p. 151–153 °C; TLC: *R*_f_ = 0.17 (ethyl acetate/*n*-hexane 70% *v*/*v*); ^1^H-NMR: δ 2.30 (4H, m, 2 × piperazine-CH_2_), 2.42 (2H, t, *J* = 7.2, COCH_2_), 2.93 (2H, m, C*H*_2_NH), 3.33 (4H, m, overlapped with the water peak, 2 × piperazine-CH_2_), 4.34 (1H, s, CH), 5.92 (2H, s, exchangable with D_2_O, NH_2_), 6.63 (1H, t, *J* = 7.2, Ar-*H*), 6.83 (1H, m, Ar-*H*), 7.25 (3H, m, overlapping signals, exchangeable with D_2_O, SO_2_N*H*CH_2_, 2 × Ar-*H*), 7.33 (4H, t, *J* = 7.6, Ar-*H*), 7.47 (5H, m, 5 × Ar-*H*); ^13^C-NMR: δ 33.4, 41.9, 45.6, 52.5, 75.6, 116.1, 117.8, 120.6, 127.9, 128.5, 129.5, 129.9, 134.4, 143.4, 147.2, 169.3; *m*/*z* (ESI positive) 479.2 [M + H]^+^.

*3-Amino-N-(3-(4-benzhydrylpiperazin-1-yl)-3-oxopropyl)benzenesulfonamide* (**5b**). Compound **5b** was obtained in 85% yield; m.p. 110–112 °C; TLC: *R*_f_ = 0.11 (ethyl acetate/*n*-hexane 50% *v*/*v*); ^1^H-NMR: δ 2.29 (4H, m, 2 × piperazine-CH_2_), 2.46 (2H, t, m, COC*H*_2_), 3.33 (6H, m, NHC*H*_2_, 2 X piperazine-CH_2_), 4.34 (1H, s, CH), 5.59 (2H, s, exchangeable with D_2_O, NH_2_), 6.99 (1H, m, Ar-*H*), 7.02 (1H, m, Ar-*H*), 7.23 (3H, t, *J* = 7.6, Ar-*H*), 7.29 (5H, m, Ar-*H*), 7.46 (5H, m, overlapping signals, exchangeable with D_2_O, CH_2_N*H*SO_2_, 4 × Ar-*H*); ^13^C-NMR: δ 33.4, 38.4, 45.8, 52.5, 75.6, 116.4, 120.7, 122.5, 126.2, 127.6, 128.2, 129.4, 141.1, 141.6, 148.3, 164.3; *m*/*z* (ESI positive) 479.2 [M + H]^+^.

*4-Amino-N-(3-(4-benzhydrylpiperazin-1-yl)-3-oxopropyl)benzenesulfonamide* (**5c**). Compound **5c** was obtained in 53% yield; m.p. 146–148 °C; TLC: *R_f_* = 0.25 (ethyl acetate/*n*-hexane 70% *v*/*v*); ^1^H-NMR: δ 2.26 (4H, m, 2 × piperazine-CH_2_), 2.48 (2H, t, *J* = 7.0, COCH_2_) 3.05 (2H, t, *J* = 7.0, NHCH_2_), 3.43 (4H, m, 2 × piperazine-CH_2_), 4.32 (1H, s, CH), 5.95 (2H, s, exchange with D_2_O, NH_2_), 6.63 (1H, d, *J* = 8.0, Ar-*H*), 7.23 (3H, m, Ar-*H*), 7.34 (5H, m, Ar-*H*), 7.45 (6H, m, overlapping signals, exchangeable with D_2_O, CH_2_N*H*SO_2_, 5 × Ar-*H*); ^13^C-NMR: δ 33.4, 38.4, 45.8, 52.5, 75.6, 117.6, 127.9, 128.5, 128.7, 129.5, 133.4, 143.4, 144.1, 169.3; *m*/*z* (ESI positive) 479.2 [M + H]^+^.

*2-Amino-N-(3-(4-(bis(4-fluorophenyl)methyl)piperazin-1-yl)-3-oxopropyl)benzenesulfonamide* (**5d**). Compound **5d** was obtained in 65% yield; m.p. 149–152 °C; TLC: *R*_f_ = 0.52 (MeOH/DCM 10% *v*/*v*); ^1^H-NMR: δ 2.26 (4H, m, 2 × piperazine-CH_2_), 2.42 (2H, t, *J* = 6.6, COCH_2_), 3.01 (2H, q, *J* = 6.6, C*H*_2_NH), 3.46 (4H, m, 2 × piperazine-CH_2_), 4.43 (1H, s, CH), 5.92 (2H, s, exchangeable with D_2_O, NH_2_), 6.63 (1H, m, Ar-*H*), 6.84 (1H, m, Ar-*H*), 7.17 (4H, m, Ar-*H*), 7.28 (1H, m, Ar-*H*), 7.37 (1H, m, exchangeable with D_2_O, SO_2_N*H*CH_2_), 7.48 (5H, m, Ar-*H*); ^13^C-NMR: δ 32.3, 38.5, 45.3, 51.3, 72.6, 116.1, 116.2 (d, ^2^*J*_C–F_ 21), 116.8, 117.8, 129.9, 130.3 (d, ^3^*J*_C–F_ 8), 134.4, 139.3, 147.2, 162.1 (d, ^1^*J*_C–F_ 242), 169.3; ^19^F-NMR: δ −115.6 (2F, s); *m*/*z* (ESI positive) 515.2 [M + H]^+^.

*3-Amino-N-(3-(4-(bis(4-fluorophenyl)methyl)piperazin-1-yl)-3-oxopropyl)benzenesulfonamide* (**5e**). Compound **5e** was obtained in 45% yield; m.p. 140–142 °C; TLC: *R*_f_ = 0.20 (MeOH/DCM 5% *v*/*v*); ^1^H-NMR: δ 2.26 (4H, m, 2 × piperazine-CH_2_), 2.46 (2H, t, *J* = 7.0, COCH_2_), 2.95 (2H, q, *J* = 7.0, CH_2_NH), 3.45 (4H, m, piperazine-CH_2_), 4.43 (1H, s, CH), 5.62 (2H, s, exchange with D_2_O, NH_2_), 6.78 (1H, d, *J* = 7.0, Ar-*H*), 6.88 (1H, d, *J* = 7.0, Ar-*H*), 6.99 (1H, s, Ar-*H*), 7.19 (5H, m, Ar-*H* ), 7.33 (1H, s, exchangeable with D_2_O, SO_2_NHCH_2_) 7.47 (4H, m, Ar-*H*); ^13^C-NMR: δ 33.3, 40.4 (overlapped with DMSO peak), 44.7, 51.3, 73.5, 112.9, 114.9, 116.2 (d, ^2^*J*_C–F_ 21), 119.0, 130.3 (d, ^3^*J*_C–F_ 8), 130.8, 140.2, 142.5, 154.2, 162.0 (d, ^1^*J*_C–F_ 242), 170.2; ^19^F-NMR: δ −115.6 (2F, s); *m*/*z* (ESI positive) 515.2 [M + H]^+^.

*4-Amino-N-(3-(4-(bis(4-fluorophenyl)methyl)piperazin-1-yl)-3-oxopropyl)benzenesulfonamide* (**5f**). Compound **5f** was obtained in 53% yield; m.p. 160–162 °C (dec.); TLC: *R*_f_ = 0.42 (MeOH/DCM 5% *v*/*v*); ^1^H-NMR: δ 2.26 (4H, m, 2 × piperazine-CH_2_), 2.45 (2H, t, *J* = 6.6, COCH_2_), 2.90 (2H, q, *J* = 6.6, CH_2_NH), 3.37 (4H, m, 2 × piperazine-CH_2_), 4.42 (1H, s, CH), 5.96 (2H, s, exchangeable with D_2_O, NH_2_), 6.63 (2H, d, *J* = 8.8, Ar-*H*), 7.02 (1H, t, *J* = 6.6, exchangeable with D_2_O, SO_2_NHCH_2_), 7.18 (4H, m, Ar-*H*), 7.42 (2H, d, *J* = 8.8, Ar-*H*), 7.48 (4H, m, Ar-*H*); ^13^C-NMR: δ 33.3, 40.4 (overlap with DMSO peak), 44.7, 52.4, 73.6, 113.6, 116.3 (d, ^2^*J*_C–F_ 21), 126.1, 129.3, 130.3 (d, ^3^*J*_C–F_ 8), 139.3, 153.4, 162.0 (d, ^1^*J*_C–F_ 242), 169.5; ^19^F-NMR: δ −115.6 (2F, s); *m*/*z* (ESI positive) 515.2 [M + H]^+^.

*(1-((2-Aminophenyl)sulfonyl)piperidin-3-yl)(4-benzhydrylpiperazin-1-yl)methanone* (**5g**). Compound **5g** was obtained in 94% yield; m.p. 161–183 °C; TLC: *R*_f_ = 0.55 (ethyl acetate/*n*-hexane 60% *v*/*v*); ^1^H-NMR: δ 1.52 (2H, m, piperidine-CH_2_), 1.71 (2H, m, piperidine-CH_2_), 2.29 (6H, m, 2 × piperazine-CH_2_, piperidine-CH_2_), 2.79 (1H, m, COCH), 3.48 (4H, m, 2 X piperazine-CH_2_) 3.57 (2H, m, piperidine-CH_2_), 4.35 (1H, s, CH), 6.06 (2H, s, exchangeable with D_2_O, NH_2_), 6.65 (1H, t, *J* = 7.2, Ar-*H*), 6.87 (1H, d, *J* = 8.4, Ar-*H*), 7.22 (2H, m, Ar-*H*), 7.33 (5H, m, Ar-*H*), 7.36 (1H, m, Ar-*H*), 7.41 (4H, m, Ar-*H*); ^13^C-NMR: δ 24.6, 27.8, 42.0, 45.8, 46.7, 48.9, 52.9, 75.7, 116.2, 118.3, 127.9, 128.6, 129.5, 130.6, 132.5, 135.0, 143.4, 148.2, 171.5; *m*/*z* (ESI positive) 519.2 [M + H]^+^.

*(1-((3-Aminophenyl)sulfonyl)piperidin-3-yl)(4-benzhydrylpiperazin-1-yl)methanone* (**5h**). Compound **5h** was obtained in 42% yield; m.p. 157–159 °C; TLC *R*_f_ = 0.26 (ethyl acetate/*n*-hexane 60% *v*/*v*); ^1^H-NMR: δ 1.57 (2H, m, piperidine-CH_2_), 1.72 (2H, m, piperidine-CH_2_), 2.29 (6H, m, 2 × piperazine-CH_2_, piperidine-CH_2_) 2.82 (1H, m, COCH), 2.49 (6H, m, 2 × piperazine-CH_2_, piperidine-CH_2_), 4.36 (1H, s, C*H*), 5.67 (2H, s, exchange with D_2_O, N*H*_2_), 6.82 (2H, m, Ar-*H*), 6.91 (1H, m, Ar-*H*), 7.26 (3H, m, Ar-*H*), 7.36 (4H, t, *J* = 7.6, Ar-*H*), 7.48 (4H, m, Ar-*H*); ^13^C-NMR: δ 24.6, 27.7, 35.6, 38.2, 47.0, 49.2, 52.5, 75.6, 114.2, 117.1, 118.7, 126.2, 127.8, 128.5, 129.5, 132.5, 143.4, 148.7, 175.2; *m*/*z* (ESI positive) 519.2 [M + H]^+^.

*(1-((4-Aminophenyl)sulfonyl)piperidin-3-yl)(4-benzhydrylpiperazin-1-yl)methanone* (**5i**). Compound **5i** was obtained in 70% yield; m.p. 132–135 °C; TLC *R*_f_ = 0.38 (ethyl acetate/*n*-hexane 70% *v*/*v*); ^1^H-NMR: δ 1.60 (2H, m, piperidine-CH_2_), 1.73 (2H, m, piperidine-CH_2_), 2.35 (6H, m, 2 × piperazine-CH_2_, piperidine-CH_2_), 2.86 (1H, m, COCH), 3.62 (6H, m, 2 × piperazine-CH_2_, piperidine-CH_2_), 4.36 (1H, s, CH), 6.10 (2H, s, exchangeable with D_2_O peak, NH_2_), 6.67 (2H, d, *J* = 8.4, Ar-*H)*, 7.23 (2H, t, *J* = 7.4, Ar-*H*), 7.36 (6H, m, Ar-*H*), 7.49 (4H, d, *J* = 7.4, Ar-*H*); ^13^C-NMR: δ) 24.5, 27.7, 42.0, 45.7, 47.3, 49.2, 52.5, 75.6, 113.6, 125.6, 127.8, 128.4, 129.4, 130.2, 143.3, 154.0, 171.3; *m*/*z* (ESI positive) 519.2 [M + H]^+^.

*(1-((2-Aminophenyl)sulfonyl)piperidin-3-yl)(4-(bis(4-fluorophenyl)methyl)piperazin-1-yl)methanone* (**5j**). Compound **5j** was obtained in 62% yield; m.p. 160–162 °C (dec.); TLC: *R*_f_ = 0.72 (ethyl acetate/*n*-hexane 70% *v*/*v*); ^1^H-NMR: δ 1.56 (2H, m, piperidine-CH_2_), 1.73 (2H, m, piperidine-CH_2_), 2.24 (4H, m, 2 × piperazine-CH_2_), 2.37 (2H, m, piperidine-CH_2_), 2.80 (1H, m, COCH), 3.56 (6H, m, 2 × piperazine-CH_2_, piperidine-CH_2_), 4.41 (1H, s, CH), 6.05 (2H, s, exchangeable with D_2_O, NH_2_) 6.66 (1H, t, *J* = 7.8, Ar-*H*), 6.88 (1H, d, *J* = 7.8, Ar-*H*), 7.18 (4H, m, Ar-*H*), 7.31 (1H, t, *J* = 7.8, Ar-*H*), 7.41 (1H, d, *J* = 7.8, Ar-*H*), 7.48 (4H, m, Ar-*H*); ^13^C-NMR: δ 24.5, 27.6, 42.0, 44.6, 45.8, 48.9, 52.0, 73.6, 116.1, 116.2 (d, ^2^*J*_C–F_ 21), 116.7, 118.2, 130.2, (d, ^3^*J*_C–F_ 9), 130.8, 135.6, 139.2, 148.1, 162.1 (d, ^1^*J*_C–F_ 242), 171.3; ^19^F-NMR: δ −115.6 (2F, s); *m*/*z* (ESI positive) 555.2 [M + H]^+^.

*(1-((2-Aminophenyl)sulfonyl)piperidin-3-yl)(4-(bis(4-fluorophenyl)methyl)piperazin-1-yl)methanone* (**5k**). Compound **5k** was obtained in 72% yield; m.p. 160–162 °C (dec.); TLC: *R*_f_ = 0.38 (ethyl acetate/*n*-hexane 70% *v*/*v*); ^1^H-NMR: δ 1.57 (2H, m, piperidine-CH_2_), 1.73 (2H, m, piperidine-CH_2_), 2.29 (6H, m, 2 × piperazine-CH_2_, piperidine-CH_2_), 2.82 (1H, m, COCH), 3.55 (4H, m, 2 × piperazine-CH_2_), 3.63 (2H, m, piperidine-CH_2_), 4.45 (1H, s, CH), 5.68 (2H, s, exchangeable with D_2_O, NH_2_), 6.77 (2H, m, Ar-*H*), 6.92 (1H, s, Ar-*H*), 7.18 (4H, m, Ar-*H* ), 7.28 (1H, m, Ar-*H*), 7.48 (4H, m, Ar-*H* ); ^13^C-NMR: δ 24.6, 27.7, 41.9, 45.7, 47.0, 49.2, 52.3, 73.5, 112.4, 114.8, 116.3 (d, ^2^*J*_C–F_ 21), 118.7, 130.3 (d, ^3^*J*_C–F_ 8), 130.6, 136.8, 139.2, 150.4, 162.0 (d, ^1^*J*_C–F_ 242), 171.4; ^19^F-NMR: δ −115.6 (2F, s); *m*/*z* (ESI positive) 555.2 [M + H]^+^.

*(1-((4-Aminophenyl)sulfonyl)piperidin-3-yl)(4-(bis(4-fluorophenyl)methyl)piperazin-1-yl)methanone* (**5l**). Compound **5l** was obtained in 60% yield; m.p. 160–162 °C (dec.); TLC: *R*_f_ = 0.42 (ethyl acetate/*n*-hexane 70% *v*/*v*); ^1^H-NMR: δ 1.56 (2H, m, piperidine-CH_2_), 1.72 (2H, m, piperidine-CH_2_), 2.26 (6H, m, 2 × piperazine-CH_2_, piperidine-CH_2_), 2.83 (1H, m, COCH), 3.54 (4H, m, 2 × piperazine-CH_2_), 3.61 (2H, m, piperidine-CH_2_), 4.45 (1H, s, CH), 6.10 (2H, s, exchangeable with D_2_O, NH_2_), 6.68 (2H, d, *J* = 8.8, Ar-*H*), 7.18 (4H, m, Ar-*H*), 7.35 (2H, d, *J* = 8.8, Ar-*H*), 7.48 (4H, m, Ar-*H*); ^13^C-NMR: δ 24.5, 27.8, 42.3, 45.7, 47.0, 49.2, 52.4, 73.5, 113.6, 116.3 (d, ^2^*J*_C–F_ 21), 119.4, 130.3 (d, ^3^*J*_C–F_ 8), 130.5, 139.2, 154.1, 162.0 (d, ^1^*J*_C–F_ 242), 171.4; ^19^F-NMR: δ −115.6 (2F, s); *m*/*z* (ESI positive) 555.2 [M + H]^+^.

#### 3.1.4. General Procedure for the Synthesis of Sulfamides **6a**–**l**

The appropriate aminobenzensulfonamides **5a**–**l** (1.0 eq) dissolved in dry DMA (5.0 mL) at 0 °C were treated with Et_3_N (1.3 eq) and freshly prepared sulfamoyl chloride until consumption of starting material was confirmed (TLC monitoring). Then the solution was quenched with slush and extracted with EtOAc (3 × 20 mL). The combined organic layers were washed with NaHCO_3_ aqueous solution. (3 × 10 mL), HCl aqueous solution 1.0 M (1 × 10 mL), brine (3 × 10 mL), dried over Na_2_SO_4_, filtered-off and concentrated under *vacuo*. The obtained residue was purified by trituration from Et_2_O to afford the titled sulfamides **6a**–**l** [19] as white solids.

*N-(3-(4-Benzhydrylpiperazin-1-yl)-3-oxopropyl)-2-(sulfamoylamino)benzenesulfonamide* (**6a**). Compound **6a** was obtained in 57% yield; m.p. 218–220 °C (dec.); TLC: *R*_f_ = 0.50 (MeOH/DCM 10% *v*/*v*); ^1^H-NMR: δ 2.28 (4H, m, 2 × piperazine-CH_2_), 2.45 (2H, t, *J* = 7.0, COCH_2_), 3.02 (2H, q, *J* = 7.0, CH_2_NH), 3.37 (4H, m, overlap with H_2_O, 2 × piperazine-CH_2_), 4.35 (1H, s, CH), 7.23 (3H, m, Ar-*H*), 7.33 (4H, t, *J* = 7.4, Ar-*H*), 7.46 (4H, d, *J* = 7.4, Ar-*H*), 7.58 (2H, s, exchangeable with D_2_O, NHSO_2_NH_2_), 7.50 (2H, m, Ar-*H*), 7.79 (1H, m, Ar-*H*), 8.02 (1H, m, exchangeable with D_2_O, SO_2_NHCH_2_) 8.81 (1H, s, exchangeable with D_2_O, N*H*SO_2_NH_2_); ^13^C-NMR: δ 33.1, 40.4, 45.5, 52.6, 75.5, 119.5, 123.0, 127.8, 128.5, 129.4, 130.0, 131.7, 136.4, 139.0, 143.9, 170.1; *m*/*z* (ESI positive) 558.0 [M + H]^+^.

*N-(3-(4-Benzhydrylpiperazin-1-yl)-3-oxopropyl)-3-(sulfamoylamino)benzenesulfonamide* (**6b**). Compound **6b** was obtained in 54% yield; m.p. 165–167 °C; TLC: *R*_f_ = 0.42 (MeOH/DCM 10% *v*/*v*); ^1^H-NMR: δ 2.29 (4H, m, 2 × piperazine-CH_2_), 2.46 (2H, t, *J* = 7.0, COCH_2_), 2.97 (2H, m, CH_2_NH), 3.44 (4H, m, 2 × piperazine-CH_2_), 4.34 (1H, s, C*H*), 7.24 (2H, m, Ar-*H*), 7.28 (2H, s, exchangeable with D_2_O, NHSO_2_N*H*_2_), 7.33 (5H, m, Ar-*H*), 7.42 (1H, m, exchangeable with D_2_O, SO_2_N*H*CH_2_), 7.49 (5H, m, Ar-*H*), 7.62 (2H, m, Ar-*H*), 9.96 (1H, s, exchangeable with D_2_O, N*H*SO_2_NH_2_); ^13^C-NMR: δ 33.4, 40.4 (overlap with DMSO peak), 45.6, 52.5, 75.6, 116.4, 120.7, 122.5, 127.8, 128.5, 129.5, 130.6, 141.1, 143.4, 145.9, 169.2; *m*/*z* (ESI positive) 558.0 [M + H]^+^.

*N-(3-(4-Benzhydrylpiperazin-1-yl)-3-oxopropyl)-4-(sulfamoylamino)benzenesulfonamide* (**6c**). Compound **6c** was obtained in 64% yield; m.p. 122–124 °C; TLC: R_f_ = 0.30 (ethyl acetate/*n*-hexane 80% *v*/*v*); ^1^H-NMR: δ 2.29 (4H, m, 2 × piperazine-CH_2_), 2.46 (2H, t, *J* = 7.0, COCH_2_), 2.92 (2H, q, *J* = 7.0, NHCH_2_), 3.43 (4H, m, 2 × piperazine-CH_2_), 4.34 (1H, s, CH), 7.22 (2H, m, Ar-*H*), 7.31 (6H, m, Ar-*H*), 7.40 (3H, m, exchangeable with D_2_O, NHSO_2_NH_2_, SO_2_NHCH_2_), 7.46 (4H, d, *J* = 7.6, Ar-*H*), 7.70 (2H, d, *J* = 8.4, Ar-*H*), 10.18 (1H, s, exchangeable with D_2_O, NHSO_2_NH_2_); ^13^C-NMR: δ 33.4, 38.4, 45.8, 52.5, 75.6, 117.6, 127.9, 128.5, 128.7, 129.5, 133.4, 143.4, 144.1, 169.3; *m*/*z* (ESI positive) 558.0 [M + H]^+^.

*N-(3-(4-(bis(4-Fluorophenyl)methyl)piperazin-1-yl)-3-oxopropyl)-2(sulfamoylamino)benzenesulfonamide* (**6d**). Compound **6d** was obtained in 52% yield; m.p. 162–164 °C; TLC: *R*_f_ = 0.42 (MeOH/DCM 10% *v*/*v*); ^1^H-NMR: δ 2.26 (4H, m, 2 × piperazine-CH_2_), 2.45 (2H, t, *J* = 6.6, COCH_2_), 3.01 (2H, q, *J* = 6.6, CH_2_NH), 3.37 (4H, m, overlap with water peak, 2 × piperazine-CH_2_), 4.43 (1H, s, CH), 7.22 (5H, m, Ar-*H*), 7.43 (4H, m, Ar-*H*), 7.60 (2H, s, exchangeable with D_2_O, NHSO_2_NH_2_), 7.65 (2H, m, Ar-*H*), 7.75 (1H, m, Ar-*H*), 8,03 (1H, m, exchangeable with D_2_O, SO_2_NHCH_2_), 8.81 (1H, s, exchangeable with D_2_O, NHSO_2_NH_2_); ^13^C-NMR: δ 32.3, 38.5, 45.3, 51.3, 72.6, 115.1, 115.4 (d, ^2^*J*_C–F_ 21), 118.4, 123.0, 130.0, 130.3 (d, ^3^*J*_C–F_ 8), 135.9, 138.2, 139.6, 161.1 (d, ^1^*J*_C–F_ 242), 169.3; ^19^F-NMR: δ −115.6 (2F, s); *m*/*z* (ESI positive) 594.0 [M + H]^+^.

*N-(3-(4-(bis(4-Fluorophenyl)methyl)piperazin-1-yl)-3-oxopropyl)-3(sulfamoylamino)benzenesulfonamide* (**6e**). Compound **6e** was obtained in 87% yield; m.p. 182–184 °C; TLC: *R*_f_ = 0.37 (MeOH/DCM 10% *v*/*v*); ^1^H-NMR: δ 2.26 (4H, m, 2 × piperazine-CH_2_), 2.46 (2H, t, *J* = 7.0, COCH_2_), 2.95 (2H, q, *J* = 7.0, CH_2_NH), 3.40 (4H, m, piperazine-CH_2_), 4.43 (1H, s, CH), 7.17 (5H, m, Ar-*H*), 7.29 (2H, s, exchangeable with D_2_O, NHSO_2_NH_2_), 7.38 (1H, m, exchangeable with D_2_O, SO_2_NHCH_2_), 7.47 (6H, m, Ar-*H*), 7.60 (1H, s, Ar-*H*), 9.96 (1H, s, exchangeable with D_2_O, NHSO_2_NH_2_); ^13^C-NMR: δ 33.3, 40.4 (overlap with DMSO peak), 44.7, 51.3, 73.5, 116.2 (d, ^2^*J*_C–F_ 21), 117.0, 121.5, 122.7, 130.3 (d, ^3^*J*_C–F_ 8), 130.8, 136.1, 139.2, 141.3, 162.0 (d, ^1^*J*_C–F_ 242), 169.3; ^19^F-NMR: δ −115.6 (2F, s); *m*/*z* (ESI positive) 594.0 [M + H]^+^.

*N-(3-(4-(bis(4-Fluorophenyl)methyl)piperazin-1-yl)-3-oxopropyl)-4-(sulfamoylamino)benzenesulfonamide* (**6f**). Compound **6f** was obtained in 35% yield; m.p. 149–152 °C; TLC *R*_f_ = 0.21 (MeOH/DCM 5% *v*/*v*); ^1^H-NMR: δ 2.26 (4H, m, 2 × piperazine-CH_2_), 2.45 (2H, t, *J* = 6.6, COCH_2_), 2.92 (2H, q, *J* = 6.6, CH_2_NH), 3.37 (4H, m, overlapped with water peak, 2 × piperazine-CH_2_), 4.43 (1H, s, CH), 7.16 (4H, m, Ar-*H*), 7.22 (2H, d, *J* = 8.8, Ar-*H*), 7.40 (2H, s, exchangeable with D_2_O, NHSO_2_NH_2_), 7.47 (5H, m, 4 × Ar-*H*, exchangeable with D_2_O, SO_2_NHCH_2_), 7.76 (2H, d, *J* = 8.8, Ar-*H*), 10.20 (1H, s, exchangeable with D_2_O, NHSO_2_NH_2_); ^13^C-NMR: δ 33.3, 40.4 (overlapped with DMSO peak), 44.7, 52.4, 73.6, 116.3, 116.6 (d, ^2^*J*_C–F_ 21), 129.4, 130.1, 130.3 (d, ^3^*J*_C–F_ 8), 139.3, 142.9, 162.0 (d, ^1^*J*_C–F_ 242), 169.5; ^19^F-NMR: δ −115.6 (2F, s); *m*/*z* (ESI positive) 594.0 [M + H]^+^.

*(1-((2-Sulfamoylaminophenyl)sulfonyl)piperidin-3-yl)(4-benzhydrylpiperazin-1-yl)methanone* (**6g**). Compound **6g** was obtained in 73% yield; m.p. 143–145 °C (dec.); TLC: *R*_f_ = 0.25 (MeOH/DCM 5% *v*/*v*); ^1^H-NMR: δ 1.56 (2H, m, piperidine-CH_2_), 1.76 (2H, m, piperidine-CH_2_), 2.40 (4H, m, 2 × piperazine-CH_2_), 2.84 (1H, m, COCH), 3.49 (4H, m, 2 × piperazine-CH_2_), 3.62 (4H, m, 2 × piperidine-CH_2_), 4.35 (1H, s, CH), 7.25 (3H, m, Ar-*H*), 7.34 (4H, m, Ar-*H*), 7.46 (4H, m, Ar-*H*), 7.71 (5H, m, overlapping signals, exchangeable with D_2_O, NHSO_2_N*H*_2_, 3 × Ar-*H*), 8.16 (1H, s, exchangeable with D_2_O, NHSO_2_NH_2_); ^13^C-NMR: δ 24.5, 27.7, 42.0, 45.8, 46.7, 48.9, 52.5, 75.6, 119.0, 123.2, 127.8, 128.5, 129.4, 129.5, 132.4, 135.5, 138.2, 143.4, 171.3; *m*/*z* (ESI positive) 598.0 [M + H]^+^.

*4(1-((3-Sulfamoylaminophenyl)sulfonyl)piperidin-3-yl)(4-benzhydrylpiperazin-1-yl)methanone* (**6h**). Compound **6h** was obtained in 44% yield; m.p. 150–152 °C; TLC: *R*_f_ = 0.39 (ethyl acetate/*n*-hexane 70% *v*/*v*); ^1^H-NMR: δ 1.59 (2H, m, piperidine-CH_2_), 1.73 (2H, m, piperidine-CH_2_), 2.27 (6H, m, 2 × piperazine-CH_2_, piperidine-CH_2_), 2.82 (1H, m, COCH), 3.56 (6H, m, 2 × piperazine-CH_2_, piperidine-CH_2_), 4.36 (1H, s, CH), 7.23 (2H, m, Ar-*H*), 7.34 (6H, m, overlapping signals, exchangeable with D_2_O, NHSO_2_NH_2_, 4 × Ar-*H*), 7.48 (6H, m, Ar-*H*), 7.57 (2H, Ar-*H*), 10.58 (1H, s, exchangeable with D_2_O, NHSO_2_NH_2_); ^13^C-NMR: δ 24.6, 27.7, 42.0, 45.8, 47.0, 49.2, 52.5, 75.6, 117.1, 121.3, 122.7, 127.9, 128.5, 129.5, 130.9, 132.6, 137.2, 141.4, 171.5; *m*/*z* (ESI positive) 598.0 [M + H]^+^.

*(1-((4-Sulfamoylaminophenyl)sulfonyl)piperidin-3-yl)(4-benzhydrylpiperazin-1-yl)methanone* (**6i**). Compound **6i** was obtained in 39% yield; m.p. 183–185 °C (dec.); silica gel TLC *R*_f_ = 0.27 (ethyl acetate/*n*-hexane 70% *v*/*v*); ^1^H-NMR: δ 1.60 (2H, m, piperidine-CH_2_), 1.73 (2H, m, piperidine-C*H*_2_), 2.35 (6H, m, 2 × piperazine-CH_2_, piperidine-CH_2_), 2.86 (1H, m, COCH), 3.62 (6H, m, 2 × piperazine-CH_2_, piperidine-CH_2_), 4.36 (1H, s, CH), 7.23 (2H, t, *J* = 7.4, Ar-*H*), 7.36 (6H, m, Ar-*H*), 7.49 (6H, m, overlapping signals, exchangeable with D_2_O, NHSO_2_NH_2_, 4 × Ar-*H*), 7.64 (2H, d, *J* = 8.4, Ar-*H*), 10.49 (1H, s, exchange with D_2_O, NHSO_2_NH_2_); ^13^C-NMR: δ 24.5, 27.7, 42.0, 45.7, 47.3, 49.2, 52.5, 75.6, 113.6, 127.8, 128.5, 129.4, 129.8, 130.3, 140.9, 142.2, 171.5; *m*/*z* (ESI positive) 598.0 [M + H]^+^.

*(1-((2-Sulfamoylaminophenyl)sulfonyl)piperidin-3-yl)(4-(bis(4-fluorophenyl)methyl)piperazin-1-yl)methanone* (**6j**). Compound **6j** was obtained in 25% yield; m.p. 142–144 °C; TLC: *R*_f_ = 0.58 (MeOH/DCM 10% *v*/*v*); ^1^H-NMR: δ 1.56 (2H, m, piperidine-CH_2_), 1.73 (2H, m, piperidine-CH_2_), 2.24 (4H, m, 2 × piperazine-CH_2_), 2.37 (2H, m, piperidine-CH_2_), 2.80 (1H, m, COCH), 3.56 (6H, m, 2 × piperazine-CH_2_, piperidine-CH_2_), 4.41 (1H, s, CH), 7.15 (3H, t, *J* = 8.4, Ar-*H*), 7.23 (2H, m, Ar-*H*), 7.45 (4H, m, Ar-*H*), 7.69 (5H, m, overlapping signals, exchangeable with D_2_O, NHSO_2_N*H*_2_, 3 × Ar-*H*), 8.82 (1H, s, exchangeable with D_2_O, NHSO_2_NH_2_); ^13^C-NMR: δ 24.5, 27.6, 42.0, 44.6, 45.8, 48.9, 52.0, 73.6, 115.9, 116.3 (d, ^2^*J*_C–F_ 21), 119.1, 123.1, 130.2, (d, ^3^*J*_C–F_ 9), 130.8, 135.6, 138.2, 139.3, 162.1 (d, ^1^*J*_C–F_ 242), 171.3; ^19^F-NMR: δ −115.6 (2F, s); *m*/*z* (ESI positive) 634 [M + H]^+^.

*(1-((3-Sulfamoylaminophenyl)sulfonyl)piperidin-3-yl)(4-(bis(4-fluorophenyl)methyl)piperazin-1-yl)methanone* (**6k**). Compound **6k** was obtained in 44% yield; m.p. 162–164 °C (dec.); TLC: *R*_f_ = 0.48 (MeOH/DCM 10% *v*/*v*); ^1^H-NMR: δ 1.57 (2H, m, piperidine-CH_2_), 1.73 (2H, m, piperidine-CH_2_), 2.29 (6H, m, 2 × piperazine-CH_2_, piperidine-CH_2_), 2.82 (1H, m, COCH), 3.55 (4H, m, 2 × piperazine-CH_2_), 3.63 (2H, m, piperidine-CH_2_), 4.45 (1H, s, CH), 7.18 (4H, m, Ar-*H*), 7.33 (3H, m, overlapping signals, exchangeable with D_2_O, NHSO_2_NH_2_, Ar-*H*), 7.48 (6H, m, Ar-*H*), 7.57 (1H, m, Ar-*H*), 9.97 (1H, s, exchangeable with D_2_O, NHSO_2_NH_2_); ^13^C-NMR: δ 24.6, 27.7, 41.9, 45.7, 47.0, 49.2, 52.3, 73.5, 116.3 (d, ^2^*J*_C–F_ 21), 118.7, 121.5, 122.7, 130.3 (d, ^3^*J*_C–F_ 8), 130.6, 136.8, 138.3, 139.3, 162.0 (d, ^1^*J*_C–F_ 242), 171.4; ^19^F-NMR: δ −115.6 (2F, s); *m*/*z* (ESI positive) 634.0 [M + H]^+^.

*(1-((4-Sulfamoylaminophenyl)sulfonyl)piperidin-3-yl)(4-(bis(4-fluorophenyl)methyl)piperazin-1-yl)methanone* (**6l**). Compound **6l** was obtained in 73% yield; m.p. 160–162 °C (dec.); TLC: *R*_f_ = 0.60 (MeOH/DCM 10% *v*/*v*); ^1^H-NMR: δ 1.56 (2H, m, piperidine-CH_2_), 1.72 (2H, m, piperidine-CH_2_), 2.26 (6H, m, 2 × piperazine-CH_2_, piperidine-CH_2_), 2.83 (1H, m, COC*H*), 3.54 (4H, m, 2 × piperazine-C*H*_2_), 3.61 (2H, m, piperidine-CH_2_), 4.45 (1H, s, CH), 7.18 (4H, m, Ar-*H*), 7.34 (2H, d, *J* = 8.4, Ar-*H*), 7.48 (6H, m, overlapping signals, exchangeable with D_2_O, NHSO_2_NH_2_, 4 × Ar-*H*), 7.65 (2H, d, *J* = 8.4, Ar-*H*), 10.29 (1H, s, exchangeable with D_2_O, NHSO_2_NH_2_); ^13^C-NMR: δ 24.5, 27.8, 42.3, 45.7, 47.0, 49.2, 52.4, 73.5, 116.3 (d, ^2^*J*_C–F_ 21), 117.5, 127.1, 129.6, 130.3 (d, ^3^*J*_C–F_ 8), 139.2, 144.7, 162.0 (d, ^1^*J*_C–F_ 242), 171.4; ^19^F-NMR: δ −115.6 (2F, s); *m*/*z* (ESI positive) 634.0 [M + H]^+^.

### 3.2. CA Inhibition

An Applied Photophysics (Leatherhead, UK) stopped-flow instrument has been used for assaying the CA-catalysed CO_2_ hydration activity [20]. Phenol red (at a concentration of 0.2 mM) has been used as indicator, working at the absorbance maximum of 557 nm, with 20 mM Hepes (pH 7.5) as buffer, and 20 mM Na_2_SO_4_ (for maintaining constant the ionic strength), following the initial rates of the CA-catalyzed CO_2_ hydration reaction for a period of 10–100 s. The CO_2_ concentrations ranged from 1.7 to 17 mM for the determination of the kinetic parameters and inhibition constants. For each inhibitor at least six traces of the initial 5–10% of the reaction have been used for determining the initial velocity. The uncatalyzed rates were determined in the same manner and subtracted from the total observed rates. Stock solutions of inhibitor (0.1 mM) were prepared in distilled-deionized water and dilutions up to 0.01 nM were done thereafter with the assay buffer. Inhibitor and enzyme solutions were preincubated together for 15 min at room temperature prior to assay, in order to allow for the formation of the E-I complex. The inhibition constants were obtained by non-linear least-squares methods using PRISM 3 and the Cheng-Prusoff equation, as reported earlier [27,28,29], and represent the mean from at least three different determinations. All CA isoforms were recombinant ones obtained in-house as reported earlier [27,28,29]. 

### 3.3. Molecular Modeling

The low resolution crystallographic structure of hCA I isoform coded by PDB ID: 4WR7 (1.5 Å resolution) [23] was used as rigid receptor in molecular docking simulations performed by the GOLD program (version 5.2.2). [25,26] Based on prior knowledge, a covalent docking protocol was established. The binding site was centered on the catalytic Zn ion and had a radius of 13 Å. For each ligand, 25 runs of the Genetic Algorithm (GA) were performed. The CHEMPLP scoring function with default parameters was used, while the GA search efficiency was increased up to 200%. Ligands were prepared for docking by means of OpenEye software. In details, ligands were sketched in VIDA (version 4.3.0) [30] and their protonation state was assigned by QUACPAC (version 1.6.3.1) [31]. Ligand energy minimization was performed with SZYBKI (version 1.8.0.1)[32] whereas the hydrogen atom was manually removed from the sulfamide zinc-binding moiety. 

## 4. Conclusions

In this study, we have reported the design and synthesis of 12 compounds bearing the sulfamide moiety as the zinc-binding-group (ZBG) and connected to a flexible tail section. All the synthesized compounds were evaluated for their inhibition potencies against the hCAs I, II, IV and IX. Almost all tested compounds showed high activity against the hCA I isoform. A SAR analysis revealed: (i) the *meta*-sulfamide fluoro-substituted constrained derivative **6k** was the most potent inhibitor against this isoform with a K_I_ value of 45.8 nM, 5.5-fold lower than the standard sulfonamide inhibitor acetazolamide (AAZ, K_I_ = 250 nM); (ii) a regioisomeric effect of the ZBG on the hCA I inhibition values was also present, and in particular the introduction of the sulfamide in *ortho*-position of the phenyl ring was detrimental for the inhibition potency; (iii) the introduction of the fluorine moiety was detrimental for the inhibition potency. Molecular modeling studies further supported SAR and provided structural explanation for the observed hCA I inhibition.

As for the hCA II, compounds **6g**–**l** were less potent when compared to their flexible analogues **6a**–**f**. The only exception was represented by the *ortho*-constrained derivatives **6g**, which was the most potent against this isoform (K_I_ = 89.8 nM). Noteworthy, the introduction of a fluorine moiety in this derivative to afford compound **6j** resulted in a 72-fold reduction of the inhibition potency, whereas the same modification on the flexible analog **6a** to afford **6d** resulted in a 2.5-fold enhancement of the inhibition potency. 

The compound **6d** was also the most active among the series in inhibiting the hCA IV isoform (K_I_ 116.7 nM). Again reduction of the flexibility, as in **6g**–**l**, proved detrimental for the inhibition potency against the hCA IV, except for the non-fluorinated *meta*-derivative **6h**. 

As for the hCA IX, the inhibition data of this isoform revealed a clearly enhancement of potency for the fluorinated compounds were compared to their non-halogenated analogs, up to full restoration of the activity for the ineffective *ortho*-derivatives (**6a** to **6d** and **6g** to **6j**). Furthermore, compound **6j** showed high selectivity for this isoform. The clear enhancement of the inhibition potency showed by these derivatives when the fluorine moiety was introduced, gave particular meaning to the role played by the fluorine atom in medicinal chemistry [33,34]. 

In conclusion, the compound series here reported showed different inhibition profiles against the various CA isoforms herein considered, thus representing a valuable source of new and valuable compounds for further development for medicinal chemistry purposes. 

## Figures and Tables

**Table 1:** Inhibition data of hCA I, hCA II, hCA IV, hCA IX with compounds **6a**–**l** and the standard sulfonamide inhibitor acetazolamide (**AAZ**) by a Stopped flow CO_2_ hydrase assay [20].

Compound		K_I_ (nM) *		
	hCA I	hCA II	hCA IV	hCA IX
**6a**	286.1	472.8	151.0	>10,000
**6b**	83.1	418.6	2359.9	1024.1
**6c**	75.4	438.9	123.9	2478.2
**6d**	659.6	188.6	116.7	735.1
**6e**	94.0	165.3	423.3	216.7
**6f**	63.2	406.3	201.6	1349.0
**6g**	604.6	89.8	314.0	>10,000
**6h**	71.4	910.7	1615.3	2682.4
**6i**	153.2	455.2	364.4	1410.8
**6j**	2750.9	6456.0	1504.9	1233.3
**6k**	45.8	753.4	1382.2	296.5
**6l**	326.1	786.0	466.6	902.3
**AAZ**	250.0	12.0	74.0	25.0

* Mean from three different assays, by a stopped flow technique (errors were in the range of ±5–10% of the reported values).
